# Effect of elastic grading on fretting wear

**DOI:** 10.1038/s41598-019-44269-1

**Published:** 2019-05-24

**Authors:** Emanuel Willert, Andrey I. Dmitriev, Sergey G. Psakhie, Valentin L. Popov

**Affiliations:** 1 Technische Universität Berlin, 10623 Berlin, Germany; 2 Institute of Strength Physics and Materials Science SB RAS, 634055 Tomsk, Russia; 3 National Research Tomsk State University, 634050 Tomsk, Russia

**Keywords:** Mechanical engineering, Structure of solids and liquids, Mechanical properties

## Abstract

We consider fretting wear in elastic frictional contact under influence of oscillations of small amplitude and investigate the question, how wear damage can be influenced by the introduction of material gradients. To achieve a general understanding we restrict our consideration to media with a power-law dependency of the elastic modulus on depth. In this case, a complete analytical solution can be found for the final worn shape. In the limiting case of small fretting oscillations we obtain a simple, closed-form asymptotic solution of the problem. We find that the optimum grading depends on the oscillation amplitude: for large amplitudes, the use of materials with a positive exponent decreases the wear volume whilst for very small amplitudes the use of graded materials with slightly negative exponent is beneficial. Especially interesting is the case of the Gibson-medium which may help avoiding both fretting wear and fretting fatigue.

## Introduction

Fretting wear and fatigue represent a considerable and longstanding problem in applications using frictional contacts subjected to vibrations as e.g. fretting of tubes in steam generators and heat exchangers^1–3^ joints in orthopaedics^4^, electrical connectors^5^, and dovetail blade roots of gas turbines^6,7^. The physical reason for the fretting is partial sliding in the vicinity of boundary of a frictional contact. It is due to the vanishing normal pressure at the contact boundary in contacts with curved surfaces^8^. Tangential oscillations without slip would cause a stress singularity at the contact boundary; hence, for any finite coefficient of friction, there will be some slip region in the vicinity of the contact boundary. This partial slip and the resulting fretting wear can only be prevented by using a contact with sharp edges. However, in this case, both normal and tangential stresses will be singular at the boundary and oscillating stresses will lead to fretting fatigue. Thus, applications with frictional contacts under vibrations should find an optimal path between Scylla of fretting wear and Charybdis of fretting fatigue.

A possible solution of the wear/fatigue dilemma may be the use of functionally graded materials (FGM). The problem of fretting wear is due to the interplay of normal and tangential stresses. The distribution of the normal stresses is, however, governed by deeper parts of the material compared with tangential stress components. Changing the elastic modulus of the surface layer of the material or – more generally – introducing material gradients could help solving the dilemma. FGM are fairly common for many biological and natural structures. They have been studied since the late 1950s^9^, initially in context of geomechanics^10^ and later in context of various engineering applications. It has been shown that FGM can provide better mechanical properties including wear and damage resistance than homogeneous materials^11,12^. Interest in FGM was enhanced by establishing new manufacturing techniques (3D printing^13^), which allow manufacturing of materials with basically arbitrary spatial distribution of mechanical properties. However, the possibility of solving the fretting wear/fatigue dilemma by using FGM has not been studied so far.

The necessary ingredients of any theory of fretting wear and fatigue are solutions of normal and tangential contact problems. For FGM with power-law and exponential dependency of elastic moduli on depth, these solutions have been found by Booker *et al*.^14,15^ and Giannakopoulos & Suresh^16,17^. Suresh *et al*. reported in a series of papers^16–19^ that the damage and failure resistance of graded glass/ceramic surfaces to normal and sliding contact or impact can be changed significantly compared with the ceramic or glass constituent. An analytical solution for the contact problem of a functionally graded coating with material properties varying as an exponential function was obtained in^20^. Two-dimensional frictionless and frictional contact problems have been studied in^21^ using the linear multi-layered model. In^22^ authors applied the linear multi-layered model for analysing the two-dimensional fretting contact of functionally graded coated half-spaces. Liu *et al*.^23^ used a similar approach to study the fretting contact of two elastic bodies under torsion. Wang *et al*. in^24^ suggested an efficient modelling approach for solving three-dimensional fretting contact involving multi-layered materials and functionally graded coatings.

Compared with other publications in the field, here we concentrate our attention on the *final shape* which develops due to fretting wear and investigate this shape for power-law graded materials in the *whole allowed interval* of the exponents of the power law. We try to find the answer to the question, if it is possible to reduce or even *completely exclude* fretting wear by introducing gradients of elastic moduli. To achieve a general qualitative understanding we confine ourselves to the case of axisymmetric contacts. Under these assumptions it occurs to be possible to provide largely analytic solutions of the fretting problem. We will show that the character of spatial distribution of elastic properties essentially influences the limiting shape of the worn profile. By correctly choosing the material gradient one can, depending on the fretting amplitude, indeed achieve a significant reduction of wear and, under some circumstances, the wear-less behaviour.

The central idea of the approach used in this paper to determine the limiting worn profile is very simple and robust. In^25^, it was shown that, if two bodies are pressed against each other and subjected to small-amplitude tangential oscillations, the worn profile develops in time tending to some limiting shape. In the follow-up paper^26^, it was argued that the limiting shape as found in^25^ is a universal one determined *solely by normal contact properties* of the medium and valid even for multiple-mode fretting. Moreover, the final worn shape obtained by using the method of dimensionality reduction (MDR)^27^ was shown to be in excellent agreement with experimental results^26^. However, at that time, only the MDR version applicable to spatially homogeneous media was available. In the meantime, Heß^28^, based on the general axisymmetric normal contact solution for power-law elastic grading by Jin *et al*.^29^, has developed the MDR to include functionally graded materials. Here we use the solutions found by Heß to analyse the limiting fretting shape of graded materials.

## Problem Formulation and Results

### Limiting profile shape in fretting wear of functionally graded bodies

The basic idea of determining the limiting shape remains the same as in the case of homogeneous media. Let us first state the problem and the main assumptions we accept in the present paper. All deformations shall be elastic, the contacting bodies shall be elastically similar (to ensure elastic decoupling) and the wear law shall be local. Apart from that we do not assume any particular wear and friction laws but only make the following very general assumptions about the local wear rate and frictional forces:

- Wear should only occur in the contact areas with non-vanishing relative displacement of surfaces (thus merely existence of tangential stresses is not enough for initiating the wear process).
- Wear should occur only in the contact areas with non-vanishing pressure.
- The law of friction (which does not necessarily have to be the Coulomb law) allows for the existence of a region of permanent stick.

As argued in^26^, already these assumptions unambiguously determine the limiting worn shape. From the existence of a region of permanent stick – assumption (c) – it follows that in this region there will be no wear – assumption (a) – and that the shape of the indenter in this region will coincide with the initial non-worn shape. Outside of the region of permanent stick, the wear can only vanish if the pressure reduces to zero – assumption (b). This means, that the no-contact condition must be fulfilled. However, as this form has to be achieved *due to wear*, the limiting profile of the indenter must exactly coincide with the form of the *free surface* (no-pressure condition!) produced by the initial indenter shape inside the permanent stick region. Thus, in the final state, in the slip region, the indenter and the elastic half space will be finally in the state of “incipient contact”. This logic does not depend on whether the material is homogeneous or heterogeneous, and it is not confined to axisymmetric contact configurations: The final worn shape is basically determined by the solution of the normal contact problem under the action of the profile defined only in the permanent stick region.

We consider a contact of an axisymmetric rigid indenter with an elastic half-space, whose Young’s modulus varies with depth according to the power law

1 $$E(z)={E}_{0}{z}^{k},\,-\,1 < k < 1,$$

where *z* is the vertical coordinate measured from the surface, *E*_0_ is an arbitrary positive constant and *k* is the exponent (*k* = 0 corresponds to a homogeneous material). Note, that the contact of two elastic bodies does not exhibit qualitatively different features, although in this case *k* must be the same for both materials.

In elastic contacts subjected to oscillations with a small amplitude Δ*u*^(0)^, there generally exists an inner area of permanent stick (with radius *c*) and the outer area (radius *a*) where there is slip at least during some part of the oscillation period.

As described above, the form of the limiting profile, given a permanent stick radius *c*, is only depending on the solution of the *normal* contact problem. In^25^, it was shown that the limiting worn profile has an especially simple form in the “MDR-space”. The MDR-formalism for axisymmetric normal contacts of power-law graded elastic bodies is detailed in the “Methods” section. The limiting profile in the “MDR-space” has the form^25^

2 $${g}_{\infty }(x)=\{\begin{array}{ll}{g}_{0}(x), & {\rm{for}}\,|x|\le c\\ d, & {\rm{for}}\,c < |x|\le a\end{array},$$

where ${g}_{0}(x)$ is the equivalent profile corresponding to the initial (non-worn) shape and *d* the indentation depth. This form guaranties that there is no wear in the area of permanent stick (because the shape is unchanged for $|x|\le c$ and thus, according to Eq. (20), for $r\le c$) and that the pressure outside the area of permanent stick vanishes (according to Eq. (21), where we have to insert $g^{\prime} =0$ starting with *x* = *c*). Moreover, from Eq. (20) follows that the difference between the worn and the non-worn profile in the 3D-domain will be

3 $${\rm{\Delta }}f(r):={f}_{\infty }(r)-{f}_{0}(r)=\frac{2}{\pi }\,\cos (\frac{k\pi }{2}){\int }_{c}^{r}\frac{{x}^{k}[d-{g}_{0}(x)]}{\sqrt{{({r}^{2}-{x}^{2})}^{1+k}}}{\rm{d}}x,\,{\rm{for}}\,c < r\le {a}_{\infty },$$

whereas the limiting contact radius after the fretting has to be determined from the condition ${\rm{\Delta }}f(r={a}_{\infty })=0.$ The total wear volume is given by the integral

4 $${\rm{\Delta }}V=2\pi {\int }_{c}^{{a}_{\infty }}{\rm{\Delta }}f(r)r\,{\rm{d}}r$$

Let us consider an initial non-worn profile of the general power-law form

5 $${f}_{0}(r)=A{r}^{n},$$

with some constant *A* and a positive exponent *n*. The transformed profile, according to Eq. (19) is given by

6 $${g}_{0}(x)=\kappa (n,k)A{|x|}^{n},\,\,\kappa (n,k):=\frac{n}{2}{\rm{B}}(\frac{n}{2},\frac{1+k}{2}),$$

where

7 $${\rm{B}}(a,b):=\frac{{\rm{\Gamma }}(a){\rm{\Gamma }}(b)}{{\rm{\Gamma }}(a+b)},$$

is the complete Beta function and Γ the Gamma function. With account of the condition

8 $$d={g}_{0}({a}_{0})$$

Eq. (3) gives the limiting shape in the worn annulus *c* < *r* < *a*_*∞*_:

9 $$\begin{array}{rcl}\frac{{\rm{\Delta }}f(r)}{d} & = & 1-\frac{2\,\cos (k\pi /2)}{\pi (1+k)}{(\frac{c}{r})}^{1+k}{}_{2}{\rm{F}}_{1}(\frac{1+k}{2},\frac{1+k}{2};\frac{3+k}{2};\frac{{c}^{2}}{{r}^{2}})\\  &  & +\,\frac{2\,\cos (k\pi /2)}{\pi (1+n+k)}{(\frac{r}{{a}_{0}})}^{n}\\  &  & \times \,[{(\frac{c}{r})}^{1+n+k}{}_{2}{\rm{F}}_{1}(\frac{1+k}{2},\frac{1+n+k}{2};\frac{3+n+k}{2};\frac{{c}^{2}}{{r}^{2}})]\\  &  & -\,{}_{2}{\rm{F}}_{1}(\frac{1+k}{2},\frac{1+n+k}{2};\frac{3+n+k}{2};1)],\end{array}$$

where we used the hypergeometric function

10 $${}_{2}{\rm{F}}_{1}(a,b;c;z):=\sum _{n=0}^{\infty }\frac{{\rm{\Gamma }}(a+n){\rm{\Gamma }}(b+n){\rm{\Gamma }}(c)}{{\rm{\Gamma }}(a){\rm{\Gamma }}(b){\rm{\Gamma }}(c+n)}\frac{{z}^{n}}{n!},\,|z|\le 1.$$

Note, that the above results are independent of the precise friction or wear laws, as long as the assumptions (a) – (c) stated in the introductory section are met. However, the control parameter in fretting is not the radius *c* of the permanent stick zone, but rather the amplitude of the tangential fretting oscillation, Δ*u*^(0)^. To establish a relation between *c* and Δ*u*^(0)^, we have to state a certain friction law. Under assumption of Coulomb’s law in local formulation with the coefficient of friction *μ*, the radius *c* of the stick area is given by^30^

11 $$g(c)=d(1-\frac{M{\rm{\Delta }}{u}^{(0)}}{\mu d}),\,{\rm{for}}\,{\rm{\Delta }}{u}^{(0)}\le {u}_{\max }^{(0)}:=\frac{\mu d}{M}.$$

Here *M* is the ratio of tangential to normal stiffness – the often so-called “Mindlin ratio”. As the rigid indenter and the elastic half-space shall be elastically similar, *M* reduces to^31^

12 $$M=\frac{2}{(1+k)(3+k)}.$$

If ${\rm{\Delta }}{u}^{(0)} > {u}_{\max }^{(0)}$, the contact enters the regime of full sliding. Most tribological systems suffering from fretting are subjected to *small* fretting amplitudes. The area of local slip will in these cases be small compared to the complete contact area and it is possible to derive an asymptotic closed-form solution for this limit from the above results. Introducing the small parameters

13 $$\varepsilon :=\frac{{a}_{\infty }-c}{c}\ll 1,\,{\varepsilon }_{a}:=\frac{{a}_{0}-c}{c}\approx \frac{M{\rm{\Delta }}{u}^{(0)}}{n\,\mu d}\ll 1,$$

carrying out expansions and neglecting all terms of second or higher order in *ε* or *ε*_*a*_, leads to the relation

14 $$\varepsilon \approx \frac{3-k}{2}{\varepsilon }_{a},$$

which, interestingly, is only depending on the power-law grading and not on the indenting profile. The total wear volume after the fretting process is in this limit approximately

15 $${\rm{\Delta }}V\approx \frac{8n{c}^{2}d}{(1-k)(5-k){(3-k)}^{2}}\,\cos (\frac{k\pi }{2}){[(3-k){\varepsilon }_{a}]}^{\frac{5-k}{2}}.$$

### Parabolic contact

Let us apply the obtained results to the “generic case” of parabolic contact with a radius of curvature *R*. The three-dimensional unworn profile in the vicinity of the contact,

16 $${f}_{0}(r)=\frac{{r}^{2}}{2R},$$

will readily give the equivalent profile

17 $${g}_{0}(x)=\frac{{x}^{2}}{R(1+k)}.$$

The initial contact radius and the radius of the permanent stick area are according to Eqs (8 and 11) equal to

18 $${a}_{0}=\sqrt{dR(1+k)},\,\,\,c=\sqrt{dR(1+k)(1-\frac{M{\rm{\Delta }}{u}^{(0)}}{\mu d})}={a}_{0}\sqrt{1-\frac{M{\rm{\Delta }}{u}^{(0)}}{\mu d}}.$$

**Figure 1:**
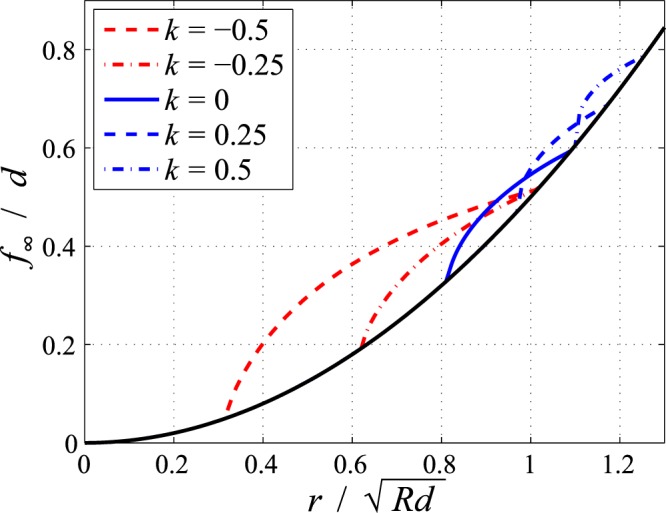
Limiting profile shape after fretting normalized for the indentation depth as a function of the normalized polar radius for a parabolic indenter with radius R for different values of exponent k of the power-law grading. Normalized fretting amplitude is 0.5. Black line denotes the unworn profile.

Figure 1 shows the limiting profile shape in normalized variables for different values of *k*. The profile has been normalised for the indentation depth and the radial coordinate for the initial contact radius in the homogeneous case, $\sqrt{Rd}$. In these variables the limiting profile only depends on *k* and the normalized fretting amplitude, which in Fig. 1 for illustration has been chosen to be ${\rm{\Delta }}{u}^{(0)}/(\mu d)=\mathrm{0.5.}$

**Figure 2:**
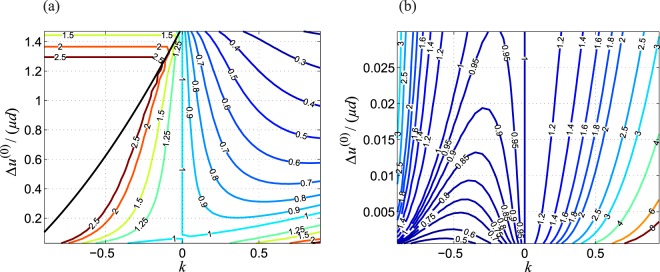
Contour isoline plots of the total worn-off volume normalized for the value in the homogeneous case for the fretting wear of a parabolic indenter on a power-law graded elastic half-space as a function of the exponent of elastic grading *k* and the normalized fretting amplitude. (**a**) Semi-analytic solution based on Eqs (4) and (9); black line denotes the transition to complete sliding. (**b**) Asymptotic solution for small fretting amplitudes according to Eq. (15).

We can easily recognize several features: first the contact radius is increasing with increasing *k*; second the slip area is propagating slower from the edge of contact inside with increasing *k*, because the Mindlin ratio *M* is decreasing with *k*; finally the worn volume seems to decrease with increasing *k*. This, however, is only a part of the complete picture which is presented in detail in Fig. 2(a,b) in form of “wear maps”. In these figures the total wear volume, normalized for the value in the homogeneous case, as a function of the two remaining parameters, the exponent *k* and the normalized fretting amplitude, is shown in contour isoline plots; the left hand side diagram (a) gives the semi-analytic solution based on the exact eqs (4 and 9), and the right hand side figure (b) shows the closed-form asymptotic solution (15).

For intermediate and large fretting amplitudes it is obviously beneficial to use a graded material with positive exponent *k*, i.e. a soft surface with a hard core. For negative *k*, i.e. hard surfaces, the wear volume is drastically increased. However, for *very small* oscillation amplitudes, it might be beneficial to use a grading with slightly negative *k*. Note, nonetheless, that the contact stresses in the case of negative elastic grading can be significantly increased if the hard surface is very thick and the soft core is isolated from carrying a relevant amount of the load. This will accelerate the wear process (although without changing the limiting profile if the indentation depth is prescribed) and may enhance fretting fatigue.

Another shape which is of interest for practical applications is the cone-profile. As it turns out, there are, however, no qualitative differences between the parabolic and conical case (we therefore won’t separately present the results for conical contact to avoid unnecessary repetition), which indicates, that the two following conclusions generally hold true, independent of the indenter shape:

- For intermediate and large fretting amplitudes, the usage of positive elastic grading (i.e. a soft surface with a harder core) reduces wear; usage of negative elastic grading (hard surface with a softer core) drastically increases wear.
- For small fretting amplitudes it may be beneficial to use (slightly) negative elastic grading.

### Some remarks on the choice of control parameters

To avoid confusion let us add some comments on the choice of fixed parameters in the considerations above.

The limiting profile after fretting wear normalized for the indentation depth will only depend on the exponent *k* of the elastic grading, the exponent *n* of the power-law profile and the ratio $c/{a}_{0}$. This is clearly indicated by Eq. (9), which has been derived under very general assumptions. However, if instead of the indentation depth *d*, for example, the normal force is prescribed, the elastic grading will also influence *d* and the “wear maps” shown above will look differently. Moreover, they will depend on more parameters (e.g. the grading thickness), which is why we chose the displacement-controlled formulation. The changes for force-control can be easily implemented based on the normal contact solution for power-law graded elastic bodies.

As said before, the radius *c* of the permanent stick area is a rather impractical choice as a control parameter, as the prescribed variable to characterize the tangential loading in fretting in most cases will be the tangential fretting amplitude. To incorporate this, we had to assume a certain friction law, involving the coefficient of friction (COF) *μ*. In the results described above, the COF was kept constant (although it easily may be influenced by the elastic grading as well^32^) because of two reasons: first, it is hard to find a systematic relation between the COF and the elastic grading and secondly we wanted to study the influence of the elastic grading on the limiting shape after fretting wear on a “fair” basis, which requires the COF to be same in the graded and in the ungraded case. However, when applying our results, one of course has to consider that the COF may be altered by the elastic grading.

### Some remarks on the possibility of wear-less behaviour

In the end we would like to give some remarks on the intriguing question of whether it is possible to achieve “no-wear” conditions in fretting contacts via functional elastic grading. From the basic assumptions stated in the introductory section it is clear, that this inevitably requires *c = a*, or in other terms, *M* = 0, i.e. the Mindlin ratio of the elastic material must vanish. This condition can be formally fulfilled in the case for the Gibson medium (i.e. *k = *1 and *ν* = 0.5), which can be shown by calculating the limit ${k}\to 1$ and $\nu \to 0.5$ in the general expressions for the normal and tangential stiffness that have been published by Heß & Popov^30^. We stress, however, that in this case the assumption of elastic similarity used throughout the present paper, is violated. Nevertheless the tangential contact stiffness due to elastic coupling with the normal contact problem should usually still be small.

Note that the use of a Gibson-Medium could be a possible solution for both problems of fretting wear and fretting fatigue as even the contacts of sharp-edged profiles with a Gibson-medium do not have any stress concentration in the vicinity of the contact boundary, which is the main reason for fretting fatigue.

## Discussion

In conclusion, we studied the final (stationary) state of fretting wear for a rigid indenter pressed into a functionally graded elastic half-space and subjected to tangential oscillations (the results obtained can straightforwardly applied for different fretting modes as well). We presented an analytical solution for the limiting indenter shape after the fretting process, from which the wear volume can be determined by simple numerical integration. For the limiting case of very small oscillation amplitudes we gave a closed-form asymptotic solution for the wear volume. Parabolic and conical contacts have been studied in detail.

We find that for intermediate and large fretting amplitudes, the usage of elastic grading with a soft surface and a harder core reduces wear (whereas grading with a hard surface and a softer core drastically increases it). For very small fretting amplitudes the usage of elastic grading with slightly positive exponent may be favourable. A possible solution to completely avoid both fretting wear and fretting fatigue may be the use of a Gibson-medium.

As stated before, our analysis is based on the assumption of purely elastic contact deformations. However, due to the stress concentration at the edge of the permanent stick area, the immediate vicinity of the stick region is prone to plastic deformations. The effect of these plastic deformations on the fretting behaviour has been studied in detail by Hu *et al*.^33^. They found that plastic deformations may allow the wear scar to continuously propagate into the contact area as the stick-slip boundary extends into the original stick area. The wear process in this case never ceases. Note, however, that increasing values of the exponent *k* reduce the stress singularity. Hence, the usage of FGM might allow to postpone or even to completely prevent this effect of plasticity. This, nonetheless, is object of further research. Moreover, in some applications the wear law might be discontinuous or non-local (e.g. due to the size of the debris particles), which would render our above findings inapplicable.

## Methods

### MDR-formalism for axisymmetric normal contacts of power-law graded bodies

Let us briefly recapitulate the solution of the normal contact problem with a graded material in the framework of MDR. Note that MDR provides an exact reformulation of the full solution of the axisymmetric contact problem in terms of a simple interpretation. No approximation or loss of information is involved. According to the general rules of the MDR, apart from the original three-dimensional axisymmetric profile $f(r)$ (*r* is the polar radius in the contact plane), a modified profile $g(x)$ is defined as derived by Heß^28^:

19 $$g(x)={|x|}^{1-k}{\int }_{0}^{|x|}\frac{f^{\prime} (r)}{\sqrt{{({x}^{2}-{r}^{2})}^{1-k}}}{\rm{d}}r,$$

where the prime denotes the first derivative. The inverse transformation reads^28^

20 $$f(r)=\frac{2}{\pi }\,\cos (\frac{k\pi }{2}){\int }_{0}^{r}\frac{{x}^{k}g(x)}{\sqrt{{({r}^{2}-{x}^{2})}^{1+k}}}{\rm{d}}x.$$

The pressure distribution in the original three-dimensional system can be calculated from

21 $$p(r)=-\,\frac{{c}_{N}}{\pi }{\int }_{r}^{a}\frac{{x}^{k}g^{\prime} (x)}{\sqrt{{({x}^{2}-{r}^{2})}^{1-k}}}{\rm{d}}x,$$

where ${c}_{N}$ is a constant depending on the material properties. If the rigid indenter is pressed into this elastic half-space by a penetration depth *d*, the contact radius *a* is determined by the condition

22 $$d=g(a)$$

Eqs (19, 21 and 22) solve the axisymmetric normal contact problem for power-law graded elastic bodies.

## Conclusions

We studied the limiting profile shape in axisymmetric contacts with power-law elastic grading under small-amplitude fretting conditions. It has been shown in the literature that the steady state (i.e. the limiting profile) exists and is universal with respect to the loading conditions, if the contact obeys the limitations of the Cattaneo-Mindlin approximation and if the wear law is local. We find that for intermediate and large fretting amplitudes, the usage of positive elastic grading (i.e. a soft surface with a harder core) reduces wear; usage of negative elastic grading (hard surface with a softer core) drastically increases wear. For small fretting amplitudes it may be beneficial to use (slightly) negative elastic grading.

## Data Availability

No additional data (other than stated in the manuscript) was produced or used for the preparation of the manuscript.
